# Hydrogen Sulfide Mediates Tumor Cell Resistance to Thioredoxin Inhibitor

**DOI:** 10.3389/fonc.2020.00252

**Published:** 2020-03-10

**Authors:** Zhimin Mao, Xiawen Yang, Sayumi Mizutani, Yanru Huang, Zhen Zhang, Hideyuki Shinmori, Kun Gao, Jian Yao

**Affiliations:** ^1^Division of Molecular Signaling, Department of the Advanced Biomedical Research, Interdisciplinary Graduate School of Medicine, University of Yamanashi, Kofu, Japan; ^2^Institute of Reproductive Medicine, School of Medicine, Nantong University, Nantong, China; ^3^Department of Biotechnology, Faculty of Life and Environmental Sciences, Graduate Faculty of Interdisciplinary Research, University of Yamanashi, Kofu, Japan; ^4^Division of Nephrology, Affiliated Hospital of Nanjing University of Chinese Medicine, Jiangsu Province Hospital of Chinese Medicine, Nanjing, China

**Keywords:** PX-12, H_2_S, sulfhydration, chemotherapy, drug resistance, Trx, albumin

## Abstract

Thioredoxin (Trx) is a pro-oncogenic molecule that underlies tumor initiation, progression and chemo-resistance. PX-12, a Trx inhibitor, has been used to treat certain tumors. Currently, factors predicting tumor sensitivity to PX-12 are unclear. Given that hydrogen sulfide (H_2_S), a gaseous bio-mediator, promotes Trx activity, we speculated that it might affect tumor response to PX-12. Here, we tested this possibility. Exposure of several different types of tumor cells to PX-12 caused cell death, which was reversely correlated with the levels of H_2_S-synthesizing enzyme CSE and endogenous H_2_S. Inhibition of CSE sensitized tumor cells to PX-12, whereas addition of exogenous H_2_S elevated PX-12 resistance. Further experiments showed that H_2_S abolished PX-12-mediated inhibition on Trx. Mechanistic analyses revealed that H_2_S stimulated Trx activity. It promoted Trx from the oxidized to the reduced state. In addition, H_2_S directly cleaved the disulfide bond in PX-12, causing PX-12 deactivation. Additional studies found that, besides Trx, PX-12 also interacted with the thiol residues of other proteins. Intriguingly, H_2_S-mediated cell resistance to PX-12 could also be achieved through promotion of the thiol activity of these proteins. Addition of H_2_S-modified protein into culture significantly enhanced cell resistance to PX-12, whereas blockade of extracellular sulfhydryl residues sensitized cells to PX-12. Collectively, our study revealed that H_2_S mediated tumor cell resistance to PX-12 through multiple mechanisms involving induction of thiol activity in multiple proteins and direct inactivation of PX-12. H_2_S could be used to predict tumor response to PX-12 and could be targeted to enhance the therapeutic efficacy of PX-12.

## Introduction

Cancer is the leading cause of death worldwide. It is, therefore, highly desirable to understand the molecular mechanisms behind the initiation and development of cancer and to find effective therapeutic interventions. Many strategies against cancer have been developed. However, the self-protective mechanisms of tumors greatly limit the efficacy of cancer therapy (1).

Redox state in tumor cells plays an important role in tumor initiation, development and therapy (2, 3). The cellular metabolic activity is associated with production of the reactive oxygen species (ROS). In normal cells, ROS is depleted by the antioxidative mechanisms. The decreased cellular defense against ROS leads to ROS accumulation, causing a situation called oxidative stress (2, 4, 5). In oxidative stress, ROS causes cell damage through modification of important molecules and activation of redox signaling pathway. In cancer cells, the metabolism is accelerated to meet the energy demand for abnormally high proliferation. The high metabolic rate leads to an increased ROS generation, which makes tumor cells under persistent oxidative stress. To escape from the stress-initiated cell injury, cancer cells develop several ways to enhance cell resistance against oxidative stress including activating alternative metabolic pathway to meet the energy demand without the large accumulation of ROS and increasing cell defense against ROS.

One of the well-documented mechanisms involved in tumor cell resistance to oxidative stress is the thioredoxin (Trx) system (6–9). The Trx system, composed of Trx reductase (TrxR), Trx, and NADPH, is one of the main thiol-dependent electron donor systems in the cells, which plays critical roles in maintaining cellular redox homeostasis and cell survival. Trx exerts its antioxidative actions via disulfide exchange. It directly scavenges ROS and plays a vital role in the control of the apoptosis signal-regulating kinase 1 (ASK1)/MAPK signaling pathway (10, 11). Besides, Trx also controls the activity of enzymes that counteract oxidative stress within cells. Trx is one of major molecules involved in the initiation and development of cancer. In many different cancers, the levels of Trx and TrxR are increased. High levels of Trx assist cancer development due to its growth-promoting and antiapoptotic functions. Trx also facilitates cancer progression through promotion of angiogenesis and metastasis. In addition, Trx contributes to tumor cell resistance to chemotherapy (9, 12, 13). Given the central roles of Trx in cancer cells, Trx has been developed as a therapeutic target to inhibit cancer growth, progression, and metastasis (14–16).

PX-12 (1-methylpropyl 2-imidazolyl disulfide) is a promising antitumor chemical that inhibit Trx activity through binding to the cysteine 73 residue of Trx (17, 18). PX-12 has shown excellent antitumor activity in both *in vitro* and *in vivo* experiments. It inhibits the growth of many different types of tumors, including human MCF-7 breast cancer and human acute myeloid leukemia cells (19, 20). Currently, PX-12 is undergoing pre-clinical trials for tumor therapy. However, factors governing tumor cell response to PX-12 are still largely unknown. To increase the therapeutic efficacy of PX-12, it is urgently needed to identify the molecules that interfere with the effects of PX-12 and to understand the mechanisms.

Hydrogen sulfide (H_2_S) is an endogenous gaseous biological mediator produced by cells expressing H_2_S synthesizing-enzymes cystathionine γ-lyase (CSE), cystathionine β-synthase (CBS), and 3-mercaptopyruvate sulfurtransferase (3-MST). H_2_S has multifaced biological actions, including antioxidative property (21–23). It scavenges ROS and enhances cell defense against oxidative stress. Many types of antioxidative machinery, such as glutathione, SOD, and catalase, is activated by H_2_S (24, 25). In many types of tumors, H_2_S-producing enzymes are upregulated, which has been recognized as a cancer-promoting factor. The endogenous H_2_S produced by tumor cells increases mitochondrial bioenergetics, accelerates cell cycle progression, stimulates cell proliferation, promotes angiogenesis and facilitates tumor cell migration and invasion (26–30). Furthermore, it enhances cell resistance to apoptosis and increases cell tolerance to several antitumor drugs (30–33).

We recently reported that H_2_S exerts its antioxidative effects through regulating the redox state of Trx (10). Also, H_2_S cleaves the disulfide bond in many molecules (10, 34, 35). These findings prompted us to speculate that H_2_S may interfere with the effects of Trx-inhibiting chemicals. The purpose of this study was to test this hypothesis.

Here, we present our data that H_2_S increases tumor cell resistance to PX-12 through multiple mechanisms, including promoting Trx reductivity, deactivating PX-12, and elevating sulfhydryl residues in proteins that competitively bind PX-12. Our study thus characterizes H_2_S as a presently unreported molecule contributing to tumor cell resistance to PX-12. Targeting H_2_S could be developed to enhance the tumor-killing efficacy of PX-12.

## Materials and Methods

### Materials

PX-12 and anti-mouse antibody against CTH were obtained from Santa Cruz Biotechnology (Santa Cruz, CA). Beta-cyano-L-Alanine (BCA) was from Cayman Chemical (Ann Arbor, MI, USA). siRNAs of CTH1 and CTH2 were purchased from QIAGEN (Tokyo, Japan). 4-acetamido-4'-maleimidylstilbene-2, 2'-disulfonic acid (AMS) was bought from Life Technologies (Eugene, OR, USA). Alexa 680 C2 maleimide was from Thermo Scientific (Rockford, IL). Anti-rabbit antibodies against Trx1 (C63C6), horseradish peroxidase (HRP)-conjugated anti-rabbit or mouse IgG were bought from Cell Signaling Technology (Danvers, MA, USA). Sodium hydrosulfide hydrate (NaHS), L-cysteine hydrochloride, DL-Propargylglycine (PAG), recombinant Trx (rTrx) and all other chemicals were from Sigma (Tokyo, Japan).

### Cells

Hepatoma G2 (HepG2), NRK52E and Hela cells were purchased from ATCC (American Type Culture Collection, Manassas, VA), which were maintained in Dulbecco's modified Eagle's medium/Ham's F-12 medium (DMEM/F-12; GIBCO-BRL, Gaithersburg, MD, USA) supplemented with 5~10% fetal bovine serum (FBS; Sigma-Aldrich, Carlsbad, CA, USA) and 1% penicillin/streptomycin/antibiotic antimycotic solution (ABAM; Sigma-Aldrich, Carlsbad, CA, USA). For experiments, cells were exposed to stimuli in the absence of FBS.

### Assessment of Cell Viability With WST Reagent

Cells were seeded onto 96-well culture plates and stimulated with various stimuli for the indicated time. WST reagent was added and allowed to react with cells for 30 min. The optical density (OD) was measured with a spectrometer at the wavelength of 450 nm. Cell viability was expressed as the percentage of OD value relative to the untreated control.

### Calcein-AM/Propidium Iodide (PI) Staining

After various treatments, cells were exposed to a mixture of Calcein-AM (green) and PI (red) solution (Dojindo, Kumamoto, Japan) for 10–20 min, and observed under a fluorescent microscope. Calcein-AM positive green cells were considered alive, while PI-positive red cells were considered dead.

### Transient Transfection

The HepG2 cells were transfected with a control siRNA or siRNA against CSE at the concentration of 20 nM using the HiPerFect transfection reagent for 36 h. Afterward, the cells were seeded onto 96- or 12-well plate, exposed to stimuli and detected for cellular expression of targeted protein and cell viability.

### Western Blot Analysis

Western blot was performed as described previously (11). Briefly, extracted proteins were separated on 10 or 12% SDS–polyacrylamide gels and electro-transferred onto polyvinylidene difluoride membranes. The membranes were blocked with 5% non-fat dry milk in phosphate-buffered saline (PBS), followed by overnight incubation with primary antibody at 4°C. Afterward, the membranes were washed and probed with HRP-conjugated anti-rabbit or anti-mouse IgG. The signals in the membrane were visualized by Chemi-Lumi One L (NacalaiTesque, Kyoto, Japan) and captured with a Fujifilm luminescent image LAS-1000 analyzer (Fujifilm, Tokyo, Japan). β-tubulin or β-actin was used as an internal loading control. Quantification of the bands was performed using ImageJ software.

### Lead Sulfide Method for Determination of H_2_S Production Capacity

H_2_S production capacity was determined according to the method described by Hine et al. (36). Briefly, H_2_S test paper was soaked in 20 mM lead acetate solution and dried. To measure H_2_S production capacity, we seeded the equal number of NRK, HepG2, or Hela cells onto 96-well culture plate. The cells were cultured in DMEM/F12 containing 10 mM L-cysteine plus 10 μM pyridoxal-5'-phosphate (PLP). The H_2_S test paper was placed directly over the 96-well plate for 24 h and allowed to react with the cell-released gaseous H_2_S. The reaction of H_2_S with lead acetate causes the formation of lead sulfide, which darkens the paper and forms a visible black-colored circle. The intensity of the circle was analyzed using the IntDen measurement in ImageJ software.

### Redox Western Analysis

Protein redox status was determined using AMS-shift assay reported by Chen et al. (37). Cellular proteins precipitated with 10% Trichloroacetic acid (TCA) were washed twice with 100% acetone and dissolved in lysis buffer (62.5 mM Tris-Cl, PH 6.8, 1% SDS). The dissolved proteins were allowed to react with 20 mM AMS at room temperature for 1 h. Afterward, the samples were subjected to Western blot analysis for the redox state of Trx under non-reducing condition.

### Red Maleimide for Detection of Sulfhydryl (-SH) Groups

This assay was modified from the previous reports (38, 39). Proteins treated with or without the indicated stimuli was precipitated with TCA, washed with acetone, redissolved in PBS and allowed to react with Alexa Fluor 680 C2 maleimide (red fluorescence at the final concentration of 2 or 5 μM) at 4°C for 2 h. The unlabeled maleimide was removed with TCA/acetone precipitation. The re-precipitated proteins were equally divided into two tubes with or without 1 mM DTT. After one hour at 4°C, the protein samples were directly applied to 0.45-μm pore size nitrocellulose membrane in a commercial dot-blot apparatus (BioRad). The signal of fluorescent maleimide in the membranes was captured with a Fujifilm image LAS-1000 analyzer (Fujifilm, Tokyo, Japan) and quantified with ImageJ software. EZ blue staining or immunoblotting of the membrane with an anti-rabbit Trx1 antibody was performed to confirm the equal loading of proteins.

### Absorption Spectroscopic Method for Detection of 2-Mercaptoimidazole

UV absorption spectra was measured to detect 2-Mercaptoimidazole (2-MI) produced by the reaction of PX-12 with NaHS using a Shimadzu UV-1800 recording spectrophotometer. Hundred micromolar PX-12 that was dissolved in a mixture of DMSO and ethanol was added into 1 mM NaHS in the volume of 1 ml PBS at room temperature for 1 h. 2-MI production was confirmed by the absorption band at 252 nm. Each measurement was repeated three times for all samples.

### Statistical Analysis

Values are expressed as mean ± SE. Comparison of two groups was made by Student's *t*-test. For multiple comparisons, one-way analysis of variance was employed. Both analyses were performed with Microsoft Excel (Microsoft, Redmond, WA, USA) or Sigma plot software (Systat Software Inc., San Jose, CA). *P* < 0.05 was considered statistically significant.

## Results

### H_2_S Regulates Cell Sensitivity to PX-12

**Figure 1:**
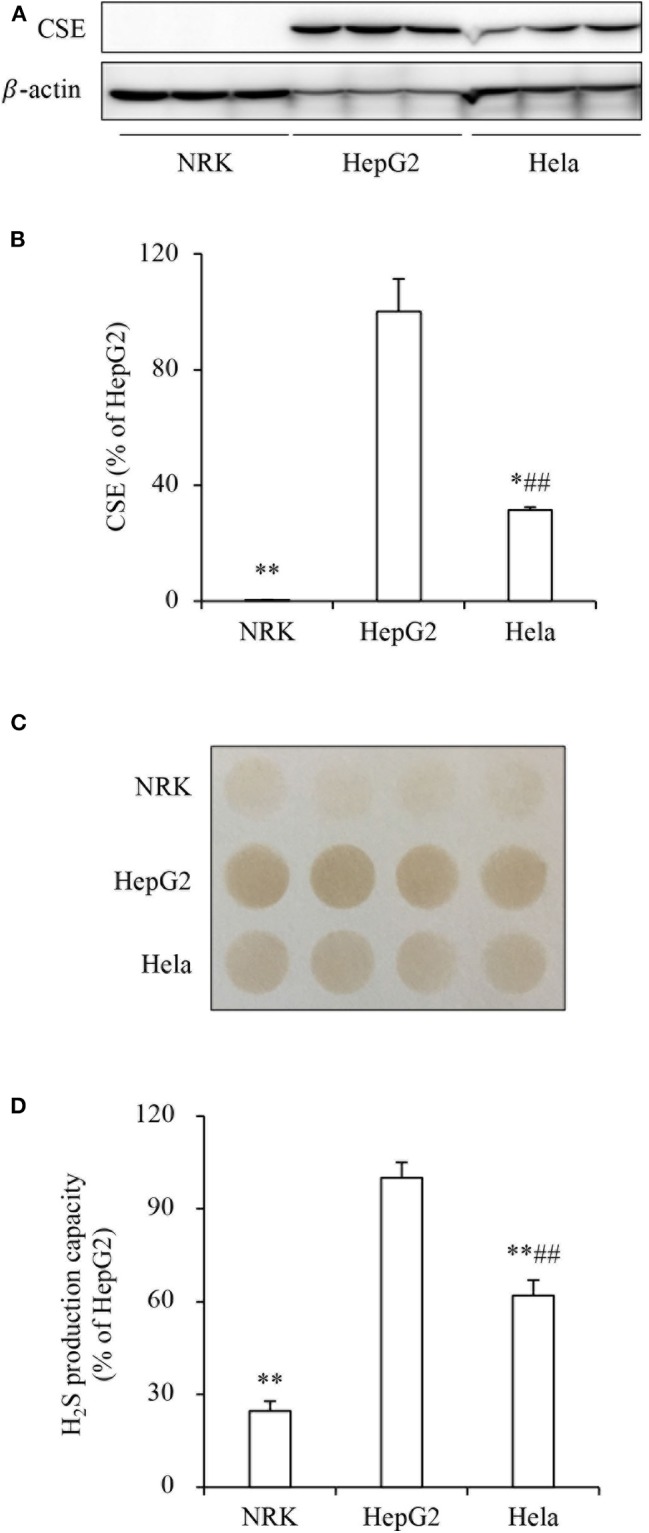
Different levels of CSE and H_2_S-producing capacity in three cell lines. **(A,B)** The protein level of CSE in NRK, HepG2, and Hela cells. The same amounts of cellular lysates from NRK, HepG2, or Hela cells were subjected to Western blot analysis for CSE **(A)**. The densitometric quantitation of the blot is shown in **(B)**. Data are expressed as percentage relative to HepG2 cells (mean ± SE, *n* = 3; ^*^P < 0.05, ^**^P < 0.01 vs. HepG2 cells; ^*##*^*P* < 0.01 vs. NRK cells). **(C,D)** Comparison of H_2_S production capacity among these cells. Cells were seeded into 96-well plate at the density of 4 × 10^5^ and cultured in growth medium supplemented with 10 mM L-cysteine and 10 μM PLP for 24 h **(C)**. The capacity of H_2_S production was determined based on the density of the black-colored circle in the test paper as described in the section of Materials and Methods. Densitometric analysis of the intensity of the black-colored circles is shown in **(D)**. Data are expressed as percentage relative to HepG2 cells (mean ± SE, *n* = 4; ^**^P < 0.01 vs. HepG2 cells; ^##^*P* < 0.01 vs. NRK cells).

To determine the role of H_2_S on tumor cell response to PX-12, we compared the effect of PX-12 among three different cell lines, NRK, HepG2 and Hela. Figures 1A,B show that these cell lines expressed different levels of H_2_S-synthesizing enzyme CSE. The amount of CSE was abundant in HepG2 cells, moderate in Hela cells and deficient in NRK cells. Consistently, H_2_S-producing capacity of these cells, as detected by using lead sulfide method, was in a direct proportion to CSE levels (Figures 1C,D). Furthermore, the level of intracellular H_2_S was indeed different between HepG2 and NRK cells as measured by using the fluorescent probe HSip-1 DA (Supplementary Figure 1).

**Figure 2:**
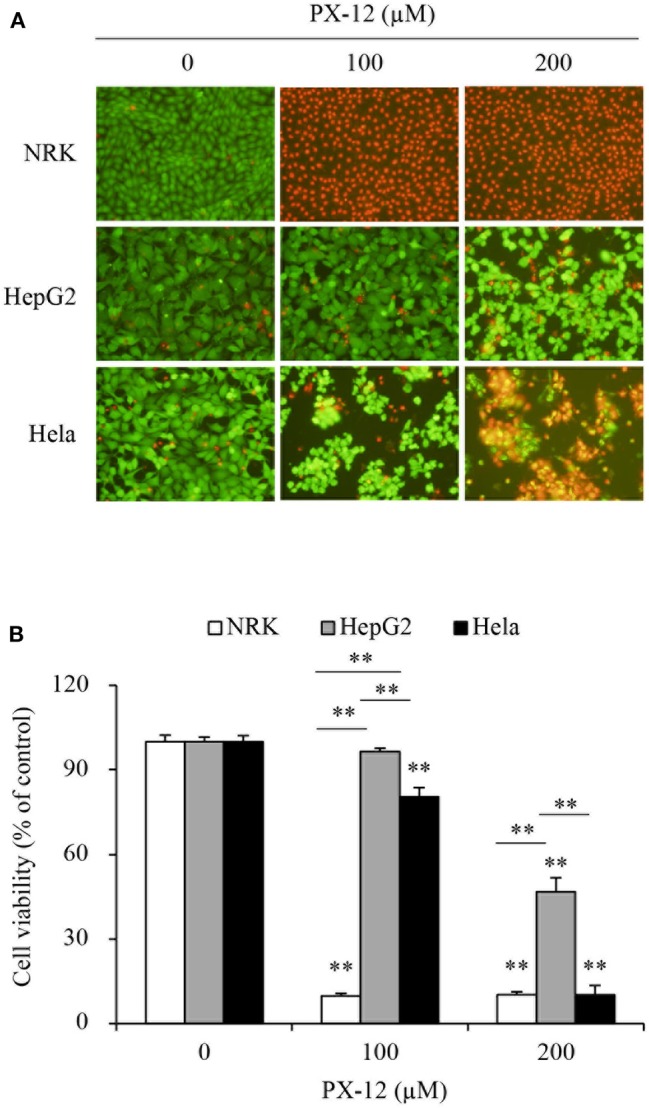
Different cell response to PX-12. **(A,B)** Effect of PX-12 on cell shape and viability. NRK, HepG2 and Hela cells were incubated with the indicated concentrations of PX-12 for 7 h. Afterward, cells were stained with Calcein-AM/PI staining (**A**, magnification: × 400) and photographed. Cell viability was evaluated by WST assay **(B)**. Data in **(B)** are expressed as the percentage of living cells against the untreated control (mean ± SE, *n* = 4; ^**^P < 0.01 vs. respective control).

We, therefore, compared the sensitivity of these cells to PX-12. Figure 2 shows that PX-12 caused a cell death in a concentration-dependent manner, which was most sensitive in CSE-deficient NRK cells and insensitive in CSE-rich HepG2 cells, while Hela cells were in the middle. These observations suggest that the cell sensitivity to PX-12 was reversely correlated to the level of CSE.

**Figure 3:**
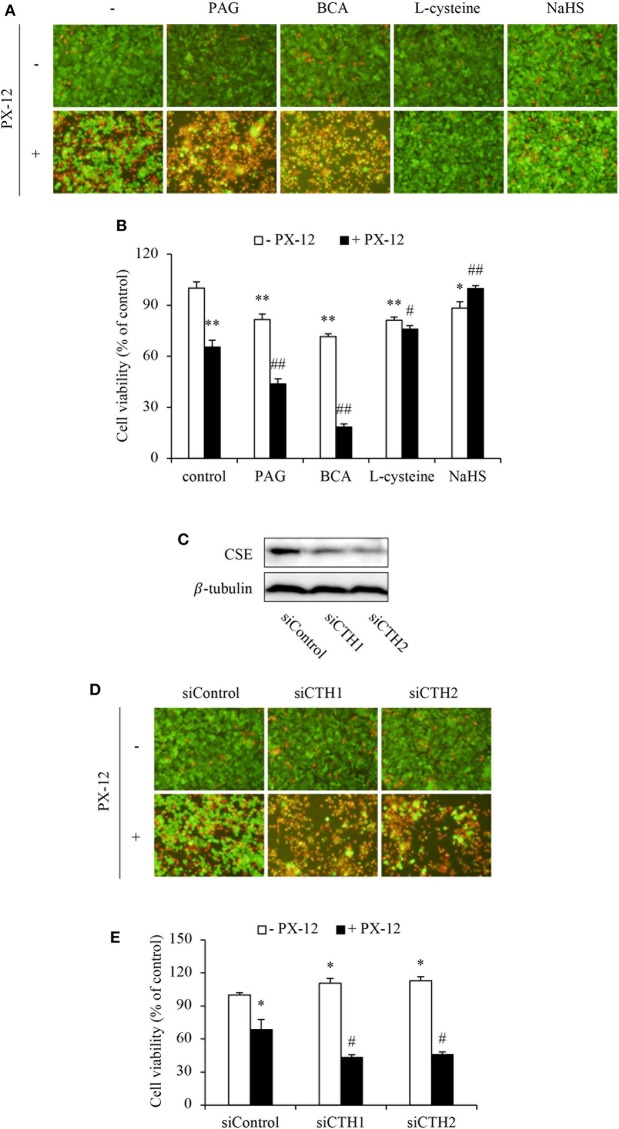
Influence of H_2_S on cell vulnerability to PX-12-induced cell death. **(A,B)** Effect of H_2_S on PX-12-induced HepG2 cell death. HepG2 cells were incubated with 200 μM PX-12 in the presence or absence of 2 mM BCA, 3 mM PAG, 2 mM L-cysteine or 1 mM NaHS for 12 h. Then, the cells were either stained with Calcein-AM/PI (**A**, magnification: × 400) or assayed for formazan formation with WST reagent **(B)**. Data in **(B)** are expressed as the percentage of living cells against the untreated control (mean ± SE, *n* = 4; ^*^P < 0.05, ^**^P < 0.01 vs. control; ^#^*P* < 0.05, ^##^*P* < 0.01 vs. PX-12 alone). **(C–E)** Effect of CSE siRNA on PX-12-induced HepG2 cell injury. The HepG2 cells were transfected with control siRNA or siRNAs targeting different sequences of CSE (siCTH1 and siCTH2) as described in Method section. The cellular lysates were extracted and subjected to Western blot analysis for CSE **(C)**. The treated cells were also seeded into 96-well plate and exposed to 200 μM PX-12 for 12 h to evaluate cell viability through Calcein-AM/PI staining (**D**, magnification: × 400) and WST assay **(E)**. Data in **(E)** are expressed as the percentage of living cells against the untreated siControl (mean ± SE, *n* = 4; ^*^P < 0.05 vs. untreated siControl; ^#^*P* < 0.05 vs. PX-12-treated siControl).

To further establish the role of H_2_S, we examined cell response to PX-12 in the presence of CSE inhibitors or H_2_S donors. Figures 3A–E show that inhibition or downregulation of CSE in HepG2 cells with chemical inhibitors (BCA and PAG) or siRNA significantly sensitized cells to PX-12. On the contrary, the supplement of HepG2 cells with H_2_S substrate L-cysteine or H_2_S donor NaHS enhanced cell resistance to PX-12. The similar resistance was also achieved in CSE-deficient NRK cells (Supplementary Figure 2). Collectively, these results indicate that H_2_S regulates cell sensitivity to PX-12.

### H_2_S Increases the Reductivity of Trx

**Figure 4:**
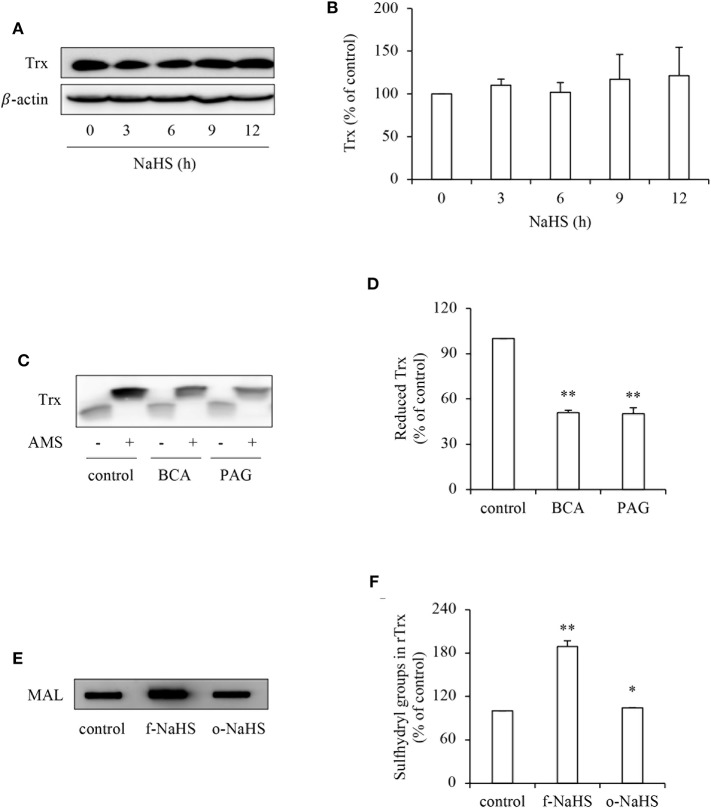
Effect of H_2_S on the redox state of Trx. **(A,B)** Effect of NaHS on Trx protein levels in HepG2 cells. HepG2 cells were exposed to 1 mM NaHS for the indicated time intervals. Cellular lysates were subjected to Western blot analysis of Trx **(A)**. Densitometric analysis of the blot in **(A)** was shown in **(B)**. Data shown are mean ± SE, (*n* = 3). **(C,D)** Effect of endogenous H_2_S on the redox state of Trx. HepG2 cells were cultured with or without 2 mM BCA or 3 mM PAG for 12 h. Cellular proteins precipitated by TCA were dissolved in lysis buffer and allowed to react with thiol-binding AMS as described in Materials and Methods. The redox state of Trx was determined through the shift of Trx bands in western blot analysis. Note the obvious reduction of AMS-labeled reduced form of Trx (upper band; **C**). Densitometric analysis of the blot in **(C)** is shown in **(D)**. Data shown are mean ± SE, (*n* = 3, ^**^P < 0.01 vs. control). **(E,F)** Effect of H_2_S on redox state of rTrx. rTrx (2 μg) was exposed to 1 mM freshly prepared NaHS solution (f-NaHS) for 2 h or solution that had been exposed to air for 2 days before the experiments (old NaHS: o-NaHS). The binding of fluorescence-labeled maleimide (MAL) was evaluated through the fluorescent intensity in dot blot **(E)**. Densitometric analysis of the blot was shown in **(F)**. Data shown are mean ± SE, (*n* = 3; ^*^P < 0.05, ^**^P < 0.01 vs. control).

To explore the mechanisms underlying the effect of H_2_S, we first examined its effect on Trx, the therapeutic target of PX-12 (17, 18). Figure 4 shows that H_2_S donor NaHS did not affect the protein expression level of Trx (Figures 4A,B), whereas it greatly affected the Trx redox state. Inhibition of CSE in HepG2 cells with BCA or PAG decreased the reduced form of Trx as revealed by AMS-shift assay (Figures 4C,D). Further experiments using rTrx show that NaHS increased the free sulfhydryl residues, as evidenced by the increased binding of thiol-reactive maleimide. This effect of NaHS disappeared after exposure of the donor solution to air to release gaseous H_2_S for 2 days, suggesting a mediating role of H_2_S (Figures 4E,F). Collectively, these results indicate that H_2_S stimulates the reductivity of Trx.

**Figure 5:**
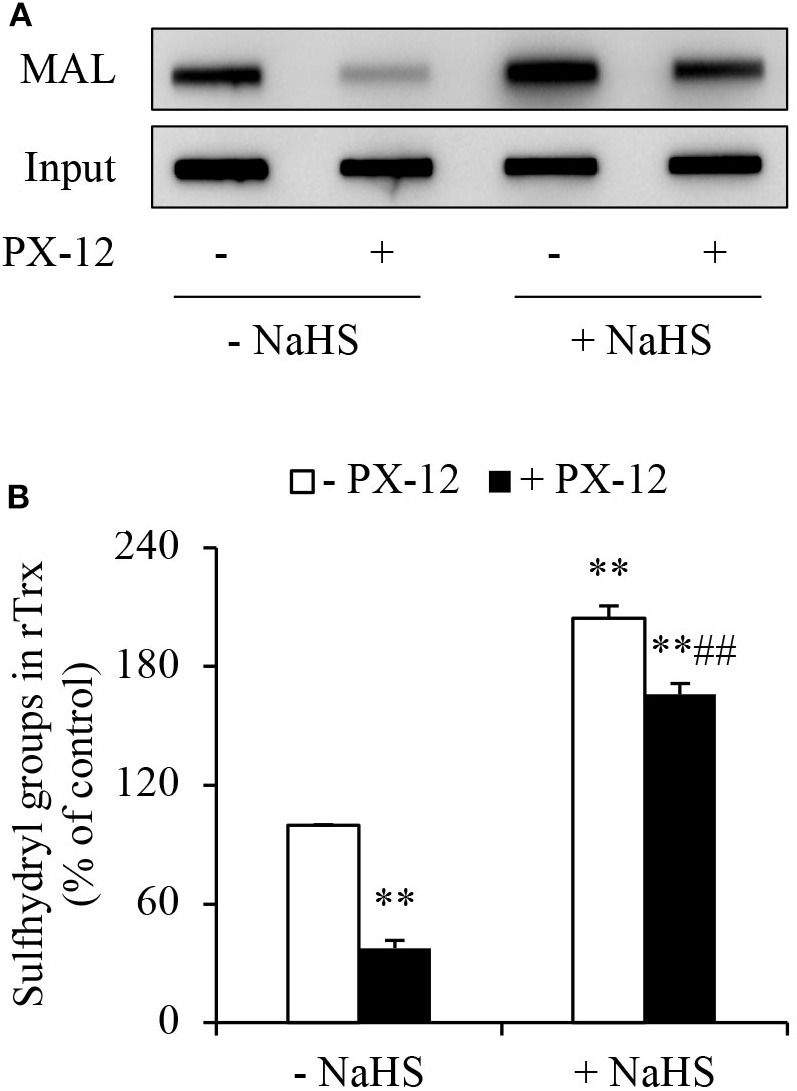
Counteracting effect of H_2_S on PX-12 binding to sulfhydryl residues in Trx. **(A,B)** Enhancement of Trx activity by H_2_S. rTrx (2 μg) was treated with 250 μM PX-12 and 1 mM NaHS for 2 h and assayed for sulfhydryl groups as described in Materials and Methods. To confirm the equal loading of rTrx, the membrane was probed for Trx using anti-Trx antibody. Note the obvious increased MAL-labeled Trx after NaHS treatment in comparison with PX-12 alone **(A)**. Densitometric analysis of the blot was shown in **(B)**. Data shown are mean ± SE, (*n* = 3; ^**^P < 0.01 vs. untreated control; ^##^*P* < 0.01 vs. PX-12 alone).

To determine whether H_2_S interfered with the effect of PX-12 on Trx, we detected free sulfhydryl residues in Trx using thiol-reactive maleimide. As expected, PX-12 potently inhibited maleimide labeling, suggesting a loss of free thiol residues in Trx. In the presence of H_2_S donor NaHS, however, the effect of PX-12 was largely abolished (Figure 5). These results indicate that H_2_S counteracts the inhibitory effect of PX-12 on Trx activity.

### H_2_S Inactivates PX-12

**Figure 6:**
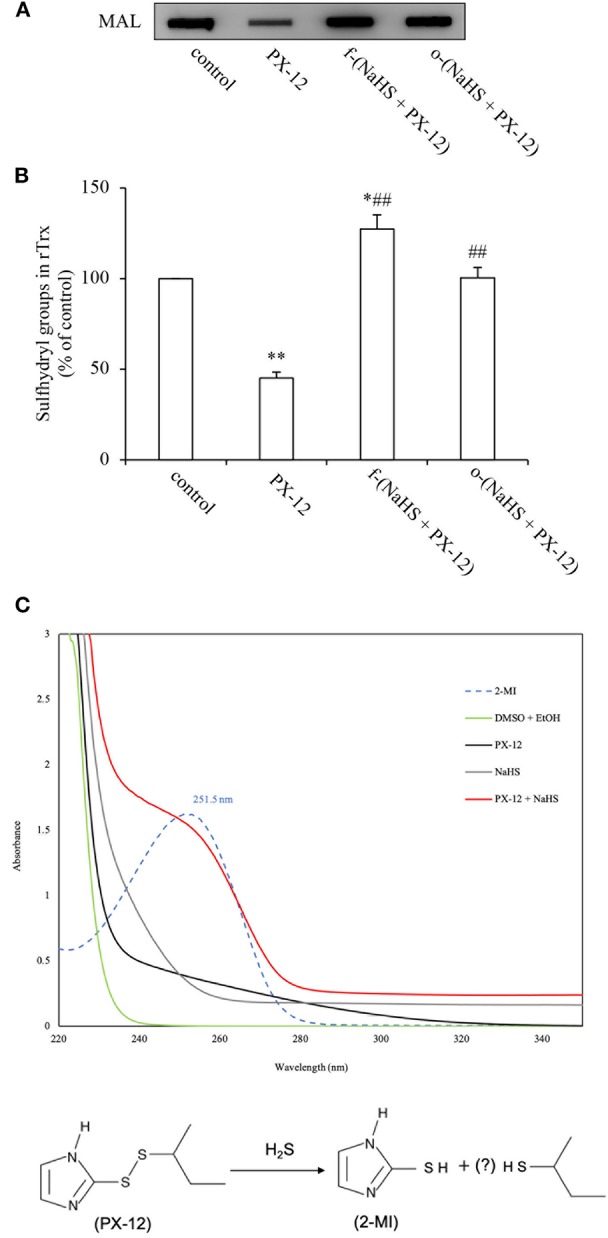
Loss of PX-12 activity after reaction with H_2_S. **(A,B)** Comparison of the effect of freshly prepared NaHS and H_2_S pre-released solution on the thiol-binding activity of PX-12. rTrx (2 μg) was treated with 250 μM PX-12, or PX-12 plus freshly prepared 1 mM NaHS for 2 h [f-(NaHS + PX-12)], or a mixture of PX-12 and NaHS solution that had been prepared 2 days before the experiment to completely release gaseous H_2_S [o-(NaHS + PX-12)]. Note that the pretreatment of PX-12 with NaHS led to a great loss of its binding ability to Trx, suggesting that H_2_S may directly inactivate PX-12 **(A)**. Densitometric analysis of the blot in **(A)** was shown in **(B)**. Data shown are mean ± SE, (*n* = 3; ^*^P < 0.05, ^**^P < 0.01 vs. control; ^##^*P* < 0.01 vs. PX-12 alone). **(C)** Production of 2-MI by the reaction of PX-12 with NaHS. 100 μM PX-12 were allowed to react with 1 mM NaHS for 1 h. The absorbance under UV-vis spectroscopic analysis was monitored. Upper panel shows the results of absorbance of different groups of chemicals. Note the appearance of a wave in NaHS plus PX-12 group (red line) at 252 nm, the peak position of the standard 2-MI (dotted line). Data shown is one of the representative of three separate experiments with similar results. Lower panel shows the scheme of potential reaction of H_2_S with PX-12. H_2_S reacted with PX-12, causing the release of 2-MI, a metabolite resulted from the cleavage of disulfide bond in PX-12.

Previous studies from our group showed that H_2_S is a reducing chemical that cleaves disulfide bond (10, 34, 35). Given that there is a disulfide bond in the structure of PX-12, which is requisite for its function, we speculated that H_2_S might directly inactivate PX-12 through reaction with disulfide bond. To test this possibility, we first examined whether pretreatment of PX-12 with H_2_S donor NaHS could lead to a loss of PX-12 activity. Figures 6A,B show that freshly prepared NaHS abolished PX-12-mediated inhibition of Trx activity. Intriguingly, this effect of NaHS was also achieved by pretreatment of PX-12 with NaHS for 2 days, a condition that H_2_S has been evaporated. This result suggests that H_2_S might directly affect PX-12 activity.

To demonstrate that H_2_S, indeed, disrupted PX-12 structure, we detected the formation of 2-MI, a metabolite resulted from the cleavage of the disulfide bond in PX-12. Figure 6C shows that standard 2-MI exhibited a peak absorbance at 252 nm under UV-vis detection (dotted line). Incubation of PX-12 with NaHS caused a formation of 2-MI, as evidenced by the appearance of a wave at the location of 2-MI (red line), which was not observed in PX-12, NaHS and dissolvent control. This result indicates that H_2_S generated by the hydrolysis of NaHS can directly deactivate PX-12.

### H_2_S Stimulates Sulfhydryl Residues in Multiple Proteins That Contribute to PX-12 Drug Resistance

**Figure 7:**
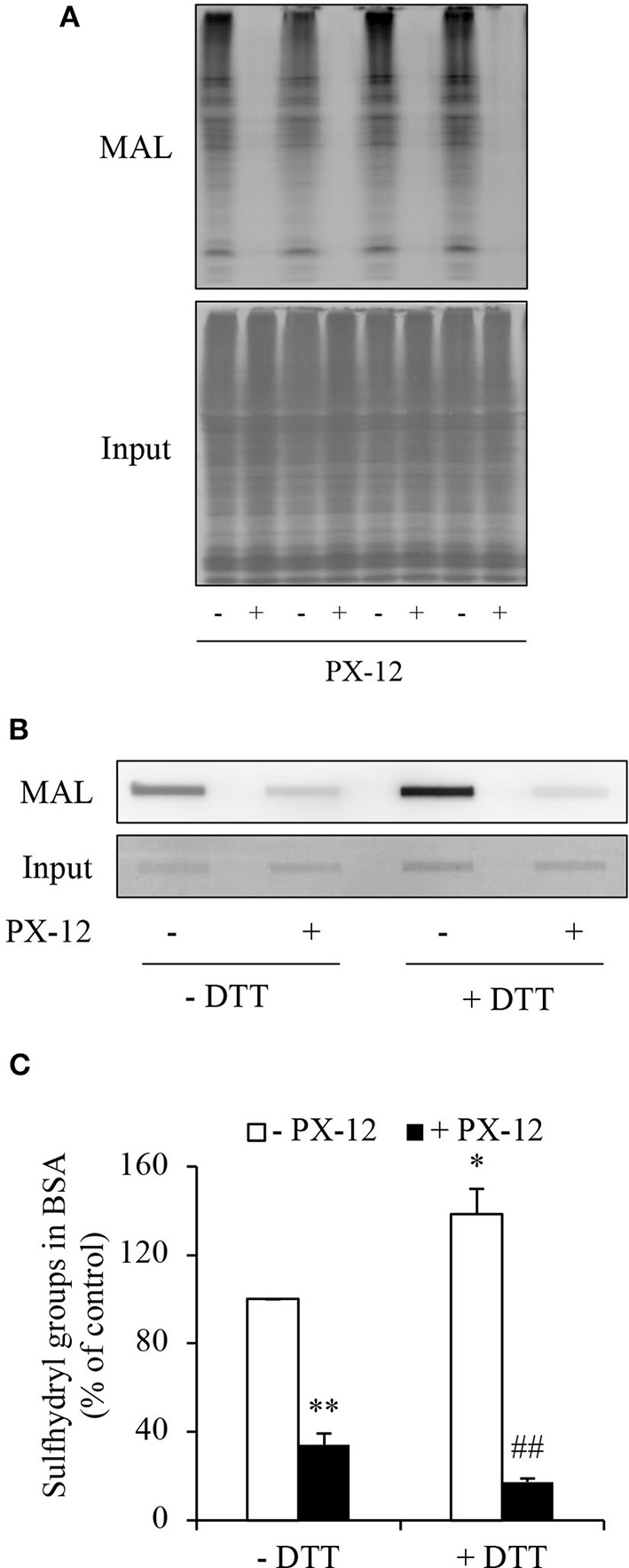
Ability of PX-12 in binding to cellular proteins and albumin. **(A)** Effect of PX-12 on MAL binding to sulfhydryl residues of cellular proteins. Cellular lysates pretreated with or without 500 μM PX-12 were exposed to thiol-reactive MAL for 2 h. The cellular proteins were separated with SDS-PAGE and blotted onto PVDF membrane **(A)**. The signal of MAL-labeled proteins was detected. Note the blocking effect of PX-12 on MAL binding to protein thiol residues (**A**, upper part). The equal loading of cellular proteins was confirmed by staining the membrane with EZ blue (**A**, lower part). **(B)** Effect of PX-12 on MAL binding to native and reduced albumin. Albumin (12.5 μg) pretreated with or without the reducing chemical DTT was exposed to 250 μM PX-12 followed by reaction with MAL, and the signal of MAL-labeled albumin was detected using dot blot. Densitometric analysis of the blot in **(B)** was shown in **(C)**. Data shown are mean ± SE, (*n* = 4; ^*^P < 0.05, ^**^P < 0.01 vs. control; ^##^*P* < 0.01 vs. PX-12 alone).

PX-12 is reported to react with thiols of Trx in a specific and irreversible way (17, 18). However, there is a report describing that PX-12 also bound tubulin and cysteine-dependent proteases (40). We, therefore, determined whether PX-12 also reacted with other cellular proteins. For this purpose, we pretreated cell lysates with PX-12 and detected its influence on thiol activity using maleimide labeling assay. Figure 7A shows that PX-12-pretreated proteins lost their binding abilities to thiol-reactive maleimide, suggesting that PX-12 was able to react with sulfhydryl groups of a wide range of cellular proteins.

Albumin is the most abundant serum protein that is produced by hepatocytes (41, 42). It is reported that albumin has active cysteine residues (43–45). We, therefore, tested whether PX-12 also reacts with albumin. Figures 7B,C show that albumin reacted with maleimide, which was potentiated by DTT, indicating the presence of sulfhydryl residues. Consistent with the notion that PX-12 also bound to proteins other than Trx, pretreatment of albumin with PX-12 caused a significant reduction in the subsequent maleimide binding. These results indicate that PX-12 reacts with sulfhydryl residues in albumin.

**Figure 8:**
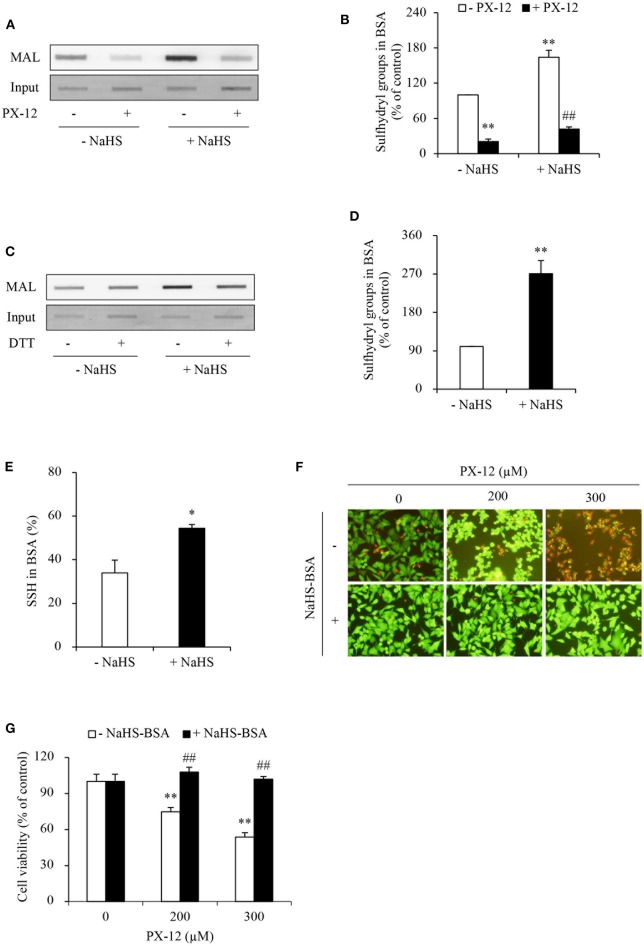
Effect of H_2_S-treated albumin on PX-12-induced cell death. **(A)** Induction of the reduced form of albumin by H_2_S. Albumin (12.5 μg) pretreated with or without 1mM NaHS were exposed to 250 μM PX-12 followed by reaction with thiol-reactive MAL for 2 h. and the signal of MAL-labeled albumin was detected using dot blot. Densitometric analysis of the blot in **(A)** was shown in **(B)**. Data shown are mean ± SE, (*n* = 4; ^**^P < 0.01 vs. control; ^##^*P* < 0.01 vs. PX-12 alone). **(C)** H_2_S induced enhancement of sulfhydryl residues and sulfhydration in albumin. Albumin (12.5 μg) treated with or without 1 mM NaHS was assayed for sulfhydration as described in the section of Materials and Methods. The samples were added to dot-blot apparatus and detected for the fluorescent signal. To confirm the equal loading of albumin, the membrane was stained with EZ blue (**C**, input). Note the obvious increased MAL-labeled Trx after NaHS treatment in comparison with control and its reduction following DTT treatment. **(D)** Densitometric analysis of the intensity of bands between control and NaHS-treated samples without DTT treatment. **(E)** Quantitative calculation of sulfhydrated albumin by NaHS. The level of sulfhydration was calculated through the fluorescence loss after DTT treatment and expressed as a percentage against the intensity of fluorescence before DTT treatment. Data in **(D,E)** are representative of four independent experiments and values are expressed in mean ± SE, (^*^P < 0.05, ^**^P < 0.01 vs. control). **(F,G)** Effect of NaHS-treated albumin on PX-12-induced cell death. HepG2 cells in 96-well plate were exposed to the indicated concentrations of PX-12 in the presence or absence of 20 mg/ml NaHS-treated albumin for 4 h. Then, the cells were stained with Calcein-AM/PI (**F**, magnification: × 400). Cell viability was determined by WST assay **(G)**. Data in **(G)** are expressed as the percentage of living cells against the respective control (mean ± SE, *n* = 4; ^**^P < 0.01 vs. control; ^##^*P* < 0.01 vs. PX-12 alone).

H_2_S has reducing activity. We have reported that H_2_S increases sulfhydryl residues of several proteins (10, 34). We, therefore, tested whether H_2_S also influence the sulfhydryl residues in albumin. Figures 8A,B show that NaHS increased sulfhydryl residues of albumin in a way similar to DTT. This effect was associated with increased albumin sulfhydration (Figures 8C–E).

Given that the increased sulfhydryl residues in albumin may compete with Trx in binding PX-12, we speculated that this mechanism might also contribute to PX-12 resistance. To confirm this hypothesis, we examined the influence of sulfhydrated albumin on PX-12-initiated cell death. Figures 8F,G show that supplement of cells with H_2_S-pretreated albumin significantly blunted the cell-killing action of PX-12.

### Blockade of Extracellular Sulfhydryl Residues Enhances Cell Sensitivity to PX-12

**Figure 9:**
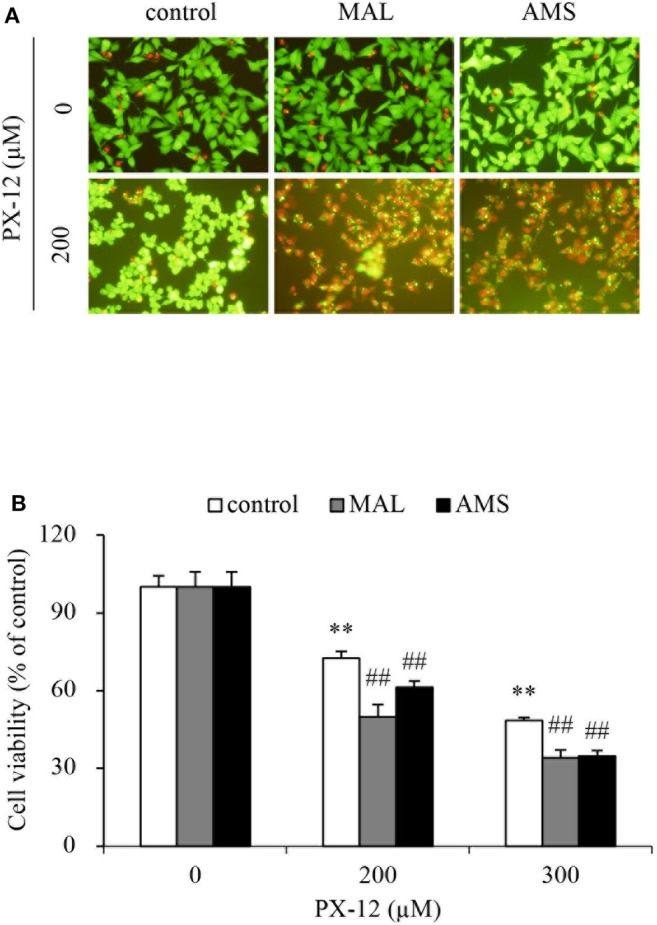
Effect of blocking sulfhydryl residues with cell membrane-impermeable alkylating chemicals on PX-12-induced cell death. **(A,B)** HepG2 cells in 96-well were pretreated with 10 μM maleimide or 2 mM AMS for 1 h, followed by exposure to the indicated concentrations of PX-12 for an additional 4 h. Then, the cells were stained with Calcein-AM/PI (**A**, magnification: × 400). Cell viability was determined by WST assay **(B)**. Data in **(B)** are expressed as the percentage of living cells against the respective control (mean ± SE, *n* = 4; ^**^P < 0.01 vs. control; ^##^*P* < 0.01 vs. PX-12 alone).

To further confirm that the off-target binding of PX-12 to the sulfhydryl residues of proteins other than Trx may also contribute to drug resistance, we blocked extracellular thiol-active residues with membrane-impermeable AMS and maleimide and examined the changes in cell response. Figure 9 shows that AMS and maleimide alone at the concentration used did not affect cell viability; however, they markedly sensitized cells to PX-12.

## Discussion

**Figure 10:**
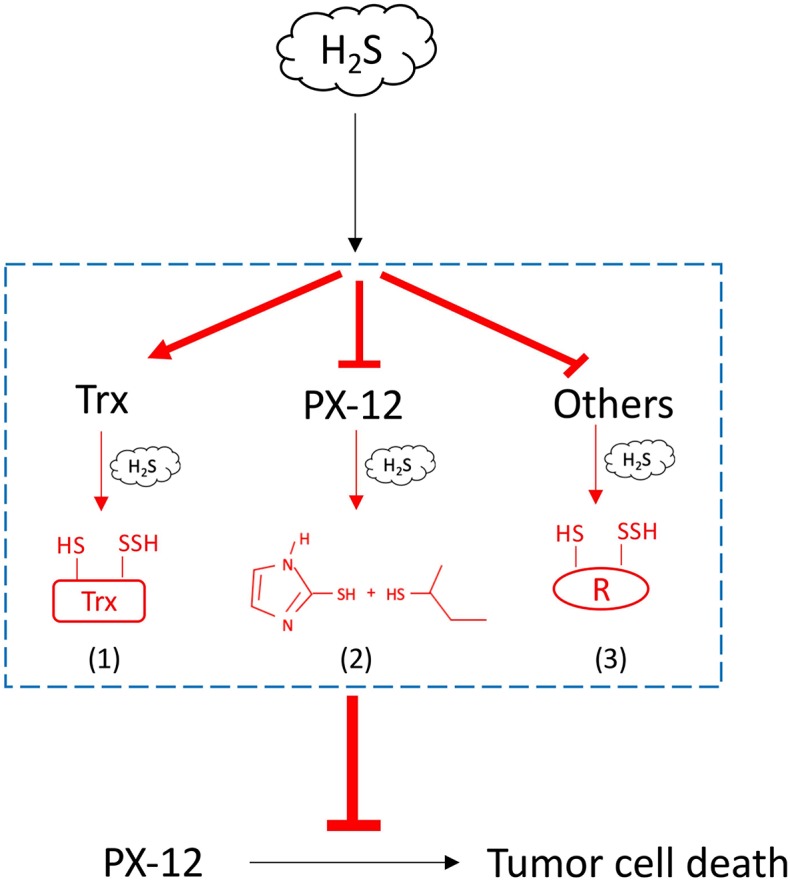
Schematic depiction of the mechanisms involved in H_2_S-mediated tumor resistance to PX-12. H_2_S increased tumor cell resistance to H_2_S through multiple mechanisms: (1) H_2_S promotes Trx in the reduced state, counteracting the thiol-inhibiting effect of PX-12; (2) H_2_S directly reacts with PX-12, leading to the cleavage of disulfide bond in PX-12 and PX-12 deactivation; and (3) H_2_S induces sulfhydryl residues in proteins that competes with Trx in binding PX-12, causing a reduced binding of PX-12 to Trx.

In this study, we characterized H_2_S as a presently unrecognized molecule influencing cell response to PX-12. Furthermore, we revealed that this effect of H_2_S was mediated through multiple mechanisms. The schematic depiction of the mechanisms has been shown in Figure 10. Given that H_2_S is produced by many types of tumors and also exists in the tumor microenvironment, H_2_S could be an important factor determining cell sensitivity to PX-12. Targeting H_2_S could be a potential way to increase the efficacy of chemotherapy.

H_2_S has been documented to increase tumor cell resistance to several drugs, such as 5-FU and oxaliplatin (30–33). In this study, we found that H_2_S underlay cell resistance to PX-12. The evidence supporting this conclusion include that: (1) cell sensitivity to PX-12 was conversely correlated with the level of H_2_S-producing enzyme CSE and H_2_S production; (2) inhibition of endogenous H_2_S sensitized cell to PX-12; and (3) supplement of cells with exogenous H_2_S enhanced cell resistance to PX-12.

Redox state is a determinant factor of tumor initiation and development. It is also the target of tumor therapy (2–4). Many antitumor drugs, such as doxorubicin, cisplatin, and bleomycin, cause tumor senescence and death through the induction of oxidative stress (3, 46–48). Previous studies have shown that ROS also mediated the tumor-killing action of PX-12; inhibition of ROS abolished its antitumor actions (49–51). Given that H_2_S regulates oxidative stress via multiple mechanisms, it is conceivable that these mechanisms could contribute to the observed effects in the current investigation.

PX-12 is an inhibitor of Trx, which inactivates Trx through interaction with the reduced thiols of Trx (17). In this study, we confirmed that the thiol activity of Trx was blocked by PX-12. Intriguingly, in the presence of H_2_S, this effect of PX-12 was abolished. The question naturally occurs as to how H_2_S prevented the effect of PX-12. Theoretically, it could be a result of its effects on Trx, PX-12, or both.

Regarding the effect of H_2_S on Trx, we have reported that H_2_S promoted the reductivity of Trx through sulfhydration (10). Here, we reconfirmed our previous finding that H_2_S increased sulfhydryl residues in Trx. This increase counteracted the action of PX-12. Besides its action on Trx, our study also revealed that H_2_S directly deactivated PX-12. This conclusion is supported by the fact that pretreatment of PX-12 with H_2_S caused a loss of PX-12 activity and a formation of 2-MI, a metabolite resulted from the cleavage of the disulfide bond in PX-12. This action of H_2_S was unexpected, but not surprising because H_2_S, as a reducing chemical, has been shown to cleave disulfide bond in many proteins (10, 34, 35).

The antitumor action of PX-12 is generally accepted to be through its specific and irreversible binding to Trx. However, there are also reports that PX-12 has off-target effects. It bound to tubulin and cysteine-dependent proteases (40). In this investigation, we found that PX-12 interacted with sulfhydryl residues of many cellular proteins, including albumin. Interestingly, our group showed that H_2_S increased the sulfhydryl residues in many proteins, including IgG and albumin (10, 34). The observations promoted us to speculate that H_2_S could enhance cell resistance through induction of sulfhydryl residues in these proteins. Our results support this idea. This is shown by the facts that supplement of cells with H_2_S-treated albumin blunted the tumor-killing action of PX-12, and that blockade of extracellular sulfhydryl residues enhanced efficacy of PX-12. Thus, induction of sulfhydryl residues in proteins other than Trx by H_2_S could be an unexpected, but important mechanism by which H_2_S increased cell resistance to PX-12.

Of note, apart from CSE, CBS and 3-MST are also involved in the production of H_2_S. In this study, their expression and roles in the tested cells have not been characterized. Given that the level of endogenous and exogenous H_2_S was closely correlated with the cell sensitivity to PX-12, it is reasonable to conclude that H_2_S is a determiant factor governing cell response to PX-12. Consistent with previous reports (52, 53), our study also indicates that CSE was the predominant enzyme for H_2_S production in HepG2 cells. The inhibition or downregulation of CSE in HepG2 cells significantly sensitized cells to PX-12. In this context, the lack of the information about CBS and 3-MST should not greatly affect our conclusion.

It is also worth mentioning that our finding could also be applicable to tumor chemotherapy targeting Trx reductase such as auranofin and pleurotin. In our previous study, we have demonstrated that Trx reductase inhibitor auranofin-induced cell death was also prevented by H_2_S (10). This effect of H_2_S could be ascribed to its promoting action on Trx reductivity. In addition, it could also be due to its induction of sulfhydryl residues in other cellular proteins as revealed in this paper.

Our study could have important basic and clinical implications. First, we identified H_2_S as a novel molecule rendering cell resistance to PX-12. In many types of tumors, increased expression of H_2_S-synthesizing enzymes has been reported (26–30, 54). Besides, tumors could also occur at an environment exposed to H_2_S, such as colon where up to millimolar H_2_S has been reported (55, 56). Moreover, H_2_S is also available from food and bacteria. This endogenous and exogenous H_2_S could hinder the therapeutic efficacy of PX-12. Strategies against H_2_S can be developed to increase efficacy of tumor therapy. Moreover, H_2_S could also be used as a potential marker predicting tumor response to PX-12.

Second, our study found that, other than Trx, PX-12 also reacted with a wide range of proteins through a thiol-disulfide exchange, including albumin, a protein that is abundant in hepatocytes and serum (41–43). Given the importance of thiol residues in protein functions, this finding should be important. It suggests that PX-12 may have a broader range of activity and applicability. It also hints that drug resistance to PX-12 should not be limited to Trx system, but also include other PX-12-reactive proteins. In this study, we showed that H_2_S significantly increased the thiol activity in a wide range of proteins, including IgG (34) and albumin. Because these proteins are far more abundant than Trx, it may play an important role in drug resistance. Thus, prevention of the off-target binding of PX-12 could be an effective way to increase therapeutic efficacy.

Third, our study provides additional evidence supporting a regulatory role of H_2_S on cysteine or disulfide bonds in multiple molecules, possibly through sulfhydration. Given thiol has been characterized as an important molecule involved in the regulation of oxidative and redox signaling, this effect of H_2_S could be an important mechanism by which H_2_S increases drug resistance. In this study, we also found that H_2_S directly deactivated PX-12. This result suggests that, in analyzing the effect and mechanisms of H_2_S on drug resistance, other than the important signaling molecules and cellular proteins, attention should also be paid to the possible direct chemical reaction between H_2_S and drugs.

Of note, our study also has limitations. We have used a relatively high, non-physiological concentration of NaHS as an exogenous H_2_S donor in some of the experiments regarding the effects of H_2_S on recombinant Trx, albumin and PX-12. However, this shortcoming should not greatly affect our conclusion about the involvement of H_2_S in cell resistance to Trx inhibitors because the similar results were also achieved through modulation of endogenous H_2_S levels. Another limitation of this study is that our finding was obtained from cultured cells. It is necessary to verify the results in animal models. This will be the direction of our future research.

Collectively, we characterized H_2_S as an important molecule governing cell response to PX-12. This effect of H_2_S involved multiple mechanisms including increasing thiol activity in Trx and in the proteins that competitively bind PX-12, as well as direct inactivation of PX-12. Our study suggests that targeting H_2_S and thiol residues could be an effective way to increase the tumor-killing efficacy of PX-12. H_2_S can be used as a marker predicting tumor cell response to PX-12.

## Data Availability Statement

All datasets generated for this study are included in the article/Supplementary Material.

## Author Contributions

ZM performed research and wrote the manuscript. XY, YH, and ZZ provided experimental assistance. SM and HS performed 2-MI detection. KG provided crucial reagents, technical assistance, and intellectual input. JY designed the study and wrote the manuscript.

### Conflict of Interest

The authors declare that the research was conducted in the absence of any commercial or financial relationships that could be construed as a potential conflict of interest.
