# Supplementary material for: Hydrogen Sulfide Mediates Tumor Cell Resistance to Thioredoxin Inhibitor
Source: Front Oncol. 2020 Mar 10;10:252. doi: 10.3389/fonc.2020.00252 (PMC7078679; doi:10.3389/fonc.2020.00252)
Supplement: Supplementary file 1 [file Image_1.pdf]

Supplementary Figure 1

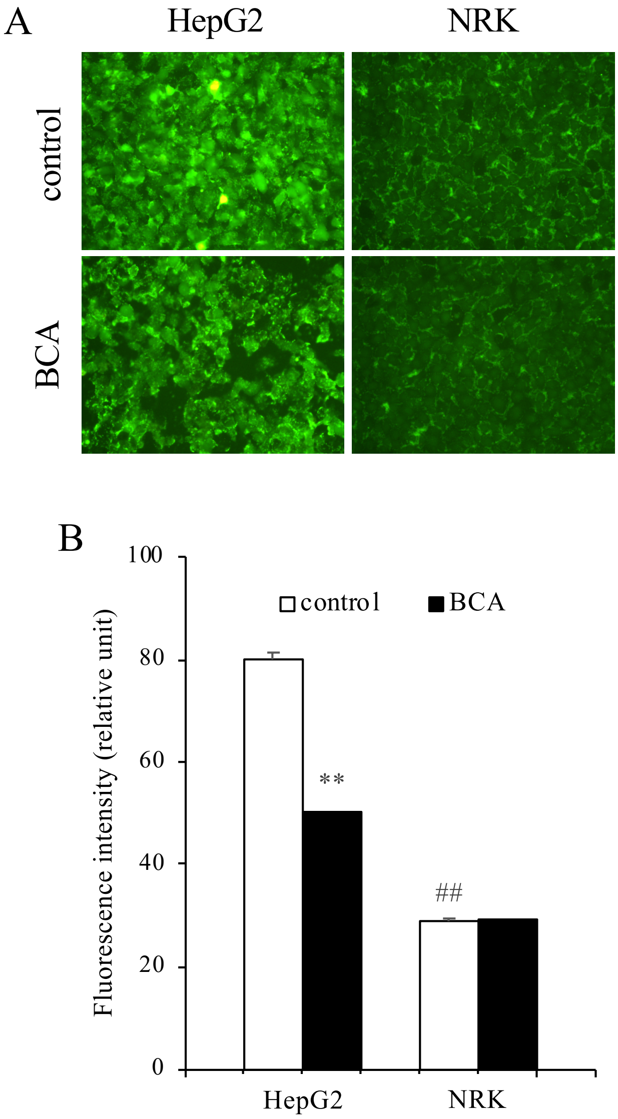

Fig. S1 Comparison of H_2S production between HepG2 and NRK cells. (A) Fluorescent images of HepG2 and NRK cells after incubation with HSip-1 DA. HepG2 and NRK cells were incubated with or without 2 mM BCA for 12 h. Afterward, cells were incubated with H_2S detection probe HSip-1 DA (5 μM) for 30 min. After washing out the probe, cells were maintained in HBSS and photographed (magnification: × 400). (B) Quantification of relative fluorescence intensity using ImageJ software. Data shown are mean ± S.E. (n = 25), ** $P < 0.01$ vs. control, ## $P < 0.01$ vs. HepG2 cells.
