# Supplementary material for: Hydrogen Sulfide Mediates Tumor Cell Resistance to Thioredoxin Inhibitor
Source: Front Oncol. 2020 Mar 10;10:252. doi: 10.3389/fonc.2020.00252 (PMC7078679; doi:10.3389/fonc.2020.00252)
Supplement: Supplementary file 2 [file Image_2.pdf]

Supplementary Figure 2

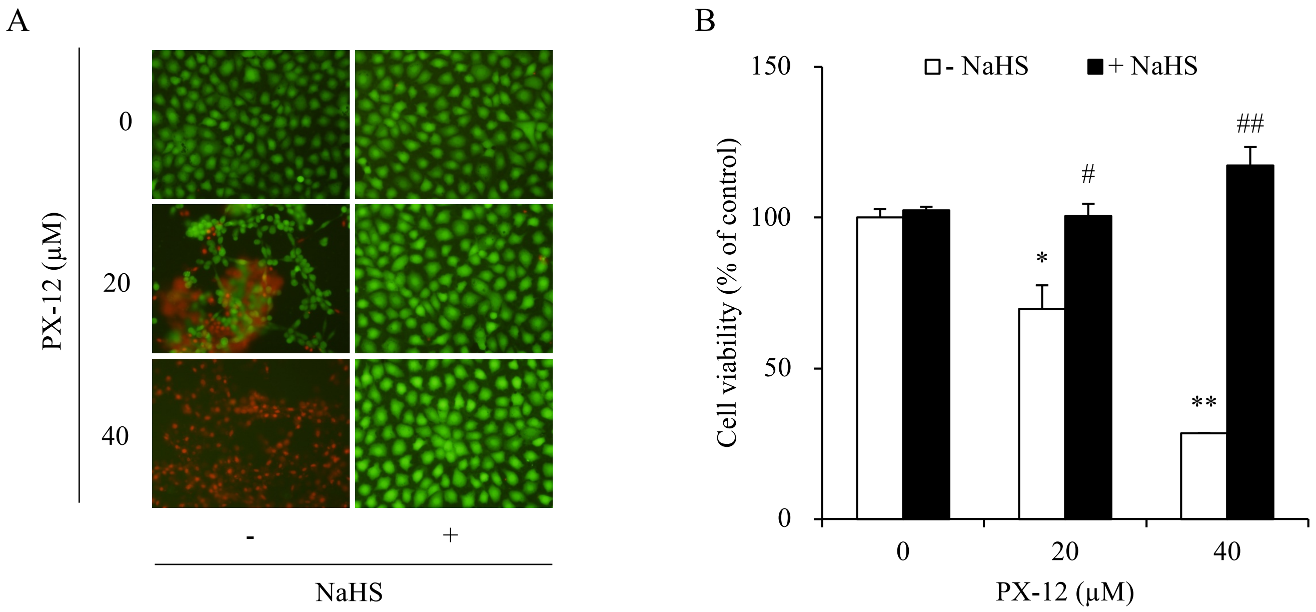

Fig. S2. H_2S protects CSE-deficient NRK cells against PX-12-induced cell death. (A) NRK cells were incubated with 20 μM or 40 μM PX-12 in the presence or absence of 1 mM NaHS for 7 h. Cells viability was determined through Calcein-AM/PI staining (magnification: × 400) and WST assay (B). Data in (B) are expressed as the percentage of living cells against the untreated control (mean ± S.E., n = 3; * $P < 0.05$, ** $P < 0.01$ vs. control; # $P < 0.05$, ## $P < 0.01$ vs. PX-12 alone).
